# The Relationship between Motivation and Burnout in Athletes and the Mediating Role of Engagement

**DOI:** 10.3390/ijerph18094884

**Published:** 2021-05-04

**Authors:** Mar Graña, Cristina De Francisco, Constantino Arce

**Affiliations:** 1 Department of Social Psychology, Basic and Methodology, University of Santiago de Compostela, 15782 Santiago de Compostela, Spain; mar.grana@rai.usc.es (M.G.); constantino.arce@usc.es (C.A.); 2 Department of Social Psychology, University of Sevilla, 41018 Sevilla, Spain

**Keywords:** motivation, burnout, engagement, athletes

## Abstract

The purpose of our research was to analyze the relationship among motivation, burnout, and engagement in sports. Five hundred athletes of both sexes from multiple sports modalities took part, with a mean age of 17.39 years (SD = 4.60). The instruments applied were as follows: Spanish versions of the Sport Motivation Scale (SMS), the Athlete Engagement Questionnaire (AEQ) and the Athlete Burnout Questionnaire (ABQ). Pearson correlations showed that motivation is negatively related to burnout and positively to engagement, while burnout and engagement are inversely related to each other. Through structural equation modeling, it was shown that engagement has a mediating role between motivation and burnout. Furthermore, there are no gender differences in this relationship, although there are differences between athletes who practice individual sports and those who practice collective sports. Encouraging high levels of self-determined motivation can help to increase athletes’ degree of engagement and protect them against burnout and sport withdrawal.

## 1. Introduction

The Self-Determination Theory (SDT) [1] within the context of sport [2,3,4] is a fundamental theoretical framework for the in-depth study of sport burnout. This macro framework, which analyses the extent to which human behavior may be volitional or self-determined, includes the sub-theory of organic integration. This sub-theory considers motivation as a continuum whose poles are non-self-determined and self-determined behaviour, identifying three fundamental types of motivation: (a) amotivation, characterized by a lack of motivation to engage in activity; (b) extrinsic motivation, defined as the motivation to engage driven by the consequences of the activity, given that it is not intrinsically reinforcing; and (c) intrinsic motivation, where the drive to engage is motivated by the internal interest, satisfaction, and reward inherent to the activity itself. In turn, extrinsic motivation can be divided into four types of motivation [1,5]: external regulation, introjected regulation, identified regulation, and integrated regulation, the latter being the most self-determined form of extrinsic motivation.

The results of studies based on the SDT in order to gain an insight into burnout in the sports context have shown that it is positively related to amotivation and negatively to intrinsic motivation [6]. The term “burnout” was first coined by German psychoanalyst Freudenberger [7]. The concept evolved through a series of major studies by authors working in the field of social psychology [8,9]. It was later applied to the context of sport, where sport burnout was defined as a chronic psychological syndrome and a three-dimensional construct: “athletes may be emotionally and physically exhausted from the psychological demands associated with a reduced sense of personal accomplishment in terms of sport abilities and achievements (…) depersonalization for athletes may be represented by the development of negative attitudes toward sport and their involvement in it”(p. 397 of [10]).

In addition to motivation, sport burnout is related to multiple negative results, such as reduced or diminished performance, and, as a last resort, withdrawal from the activity [6]. On the other hand, the greatest concern expressed by a number of researchers in their systematic study of burnout [11] is the relationship between the syndrome and the withdrawal from sporting activities. Following the principles of positive psychology, organizational psychologists interested in the study of burnout [12] have suggested that engagement is the conceptual opposite of burnout, advocating for engagement as the best method for its prevention [13]. In contrast, other researchers have posited that burnout and engagement are independent and conceptually different constructs [14]. From this perspective, a single athlete may, to a certain degree, experience both sensations simultaneously. Whatever the case, researchers agree that engagement fits within the framework of positive psychology, considering the most favorable aspects of human life and relegating to second place those less favorable aspects of the human life cycle [15,16].

Initially, engagement was considered as a means of ensuring that the members of an organization were committed to their workplace roles [17]. Years later it was defined as a positive mental state of work-related fulfilment, characterized by a sense of vitality, dedication, and absorption [12,18]. Rather than a specific momentary state, engagement refers to a deeper, longer-lasting affective–cognitive state. Following other authors [19], engagement in sport is characterized by four, rather than three, dimensions: (a) confidence, defined as the belief in the capacity to reach a high level of performance and the desired goals; (b) vigor, namely the sensation of physical and mental vitality; (c) dedication, reflected in the desire to channel time and efforts into achieving the goals that the individual considers to be of importance; and (d) enthusiasm, related to the emotions and high levels of enjoyment.

Designing and programming interventions that boost engagement, thus supporting motivation and protecting athletes against burnout, have acquired a particular relevance in the field of sport psychology. This study analyses the role of motivation in the appearance of burnout and engagement, whilst at the same time considering the role of engagement as a form of mediation in the relationship between athletes’ motivation and burnout. It was expected that motivation would be negatively related to burnout and positively related to sport engagement. Likewise, and based on a review of existing literature, an inverse relationship was predicted between burnout and engagement in the sample.

## 2. Materials and Methods

### 2.1. Design and Participants

A retrospective ex post facto design was carried out with a single group and multiple measurements. A large group of participants was selected in order to be able to have all the possible values of the “dependent” variable (burnout) and also a great variety of scores in the rest of the variables [20].

Five hundred Spanish federated athletes took part in the study. They were selected via an intentional nonprobability sampling method and came from 24 different sports, seven of which were collective sports (66%) and the remainder from individual sports (34%). The target population was federated competitive athletes in Spain ranging between 12 and 29 years old. The average age was 17.39 years (SD = 4.60). As for the breakdown by sex, 74% were men and 26% women. The participants stated that they had been practicing their sport for at least 1 year (M = 7.65, SD = 4.85) and trained an average of more than 3 times per week (M = 3.67, SD = 1.51) for at least 1 hour per session (M = 1.95, DT = 0.51).

### 2.2. Measures and Procedures

Sport motivation was assessed via the Spanish adaptation [21,22] of the SMS [23,24]. This scale comprises 28 items divided into 7 subscales (with a distribution of four items per factor): amotivation, external regulation, introjected regulation, identified regulation, intrinsic motivation to know, intrinsic motivation to accomplish, and intrinsic motivation to experience stimulation. The response format was a 7-point Likert-type scale that ranged from “completely untrue of me” (1) to “completely true of me” (7). Various studies have confirmed the reliability and validity of this instrument within the context of Spanish sports [21,22]. The global motivation index was calculated following the procedure posited by Vallerand [25], which has also been used in numerous research projects [26,27,28]:

((2 × (IM to know + IM to accomplish things + IM to experience stimulation)/3) + Identified Regulation) − (((External Regulation + Introjection)/2) + (2 × Amotivation))(1)

where IM is Intrinsic Motivation.

The Spanish version [29] of the Athlete Engagement Questionnaire (AEQ) [30] was used to assess sport engagement. This version of the questionnaire is made up of 16 items grouped proportionally into 4 factors: confidence, vigor, dedication, and enthusiasm. The response format was a 5-point Likert-type scale ranging from 1 (hardly ever) to 5 (almost always). The adaptation displayed a good fit of the model, as well as an internal consistency higher than that recommended by Nunnally [31]: α > 0.70. The reliability of the original version of this instrument was proven in a later study [30] (study 3), as was that of the Spanish version of the instrument, which also yielded satisfactory results in terms of validity and reliability [29,32].

The Spanish adaptation [33] of the Athlete Burnout Questionnaire (ABQ) [34,35] was used to assess sport burnout. It consists of 15 items grouped into 3 factors, each with 5 items: physical and emotional exhaustion (PEE), a reduced sense of accomplishment (RSA), and sport devaluation (SD). The adaptation displayed a good fit of the model, obtaining acceptable levels of reliability as well as evidence of convergent and discriminant validity [36]. The response format was a 5-point Likert-type scale: (1) hardly ever, (2) rarely, (3) sometimes, (4) often, and (5) almost always.

Contact with the clubs that participated in the research project was initially done by telephone. It included an explanation of the objectives, expressed interest in their participation, and requested a face-to-face meeting in order to provide further details. Participation in the study was very high: the response rate of sports organizations requested was 100%. In most cases, the person contacted was a member of the management board or a coach. The exceptions were a sport psychologist and the headmaster of a subsidized private school.

The questionnaires were completed mainly in the dressing rooms, press rooms, or board rooms located in the sport facilities during the training sessions conducted by the members of the research team. Again, the exception was the school, where the lecture theatre was used. Prior to handing out the booklets containing the questionnaires (in the order AEQ, ABQ, and SMS), a standard presentation was given, outlining the objectives of the study. Likewise, the participants were informed that the responses were anonymous, and an appeal was made for their complete sincerity. Finally, they were thanked for taking part in the research project. The athletes or their coaches (in the case of minors) signed the corresponding informed consent forms. The study was approved with the code CE061810 by the ethics committee of the university where one of the researchers worked.

### 2.3. Data Analysis

The statistical analyses were carried out using version 25 of IBM SPSS Statistics. They began with an exploratory analysis of the athletes’ responses to the questionnaire items in order to identify any missing or outlier values and their respective replacements or substitutions. The descriptive statistics of the study variables were then calculated (motivation, engagement, and burnout), as well as the Pearson correlation coefficients between the three constructs. The third stage consisted of identifying a path analysis model, taking motivation as the exogenous variable, burnout as the endogenous variable, and engagement as the variable that mediates between the two. The direct, indirect, and total effects of the model were calculated with version 22 of Amos Graphics statistics software. Finally, a z-test was used to compare the direct effects between male and female athletes and between those practicing collective and individual sports.

## 3. Results

### 3.1. Prior Analyses

An explanatory analysis was made of the athletes’ responses to the items included on the three questionnaires used for the purpose of the study in order to determine the existence of missing values or potentially outlier values. Very few items with missing values were identified (less than 1%), and in all cases, they were replaced with the athlete’s most frequent response to the items included in the factor that the item belonged to. No outlier values were identified.

### 3.2. Description of the Responses

Table 1 shows the descriptive statistics (mean, standard deviation, skewness, and kurtosis) for the factors corresponding to SMS, AEQ, and ABQ, as well as their totals.

Mean values for SMS factors ranged between 5.38 (IM stimulation) and 2.81 (amotivation). The highest standard deviation was noted in the external regulation factor (1.53) and the lowest in the amotivation factor (1.19). Skewness was negative for all factors except external regulation (0.04) and amotivation (0.79). Kurtosis was negative in the majority of factors, with the exception of IM to experience stimulation (0.13) and amotivation (0.56).

In the case of the questionnaire on sport engagement, enthusiasm registered the highest mean value (4.60) and confidence the lowest (3.75). The standard deviation ranged between 0.77 (dedication) and 0.67 (vigor). Negative skewness was observed in all four factors. Confidence (−0.13) was the only factor with negative kurtosis.

Finally, mean factor values for the ABQ ranged between 2.54 (RSA) and 1.71 (SD). The highest standard deviation was noted for the PEE factor (1.49) and the lowest for RSA (0.69). Positive skewness was observed for all three factors, and kurtosis was negative in the case of RSA (−0.02) and positive in the other two.

AEQ and ABQ totals were calculated from the sum of the scores for their corresponding factors, divided by the number of factors included in the questionnaire.

### 3.3. The Relationship between Motivation, Engagement, and Burnout

As was to be expected, given previous research in this field, motivation displayed a positive relationship with engagement (r = 0.38, *p* < 0.001) and a negative one with burnout (r = −0.46, *p* < 0.001), as did engagement with burnout (r = −0.55, *p* < 0.001).

In turn, Table 2 shows the Pearson correlations between the seven levels of motivation, which associate the SMS model [24] with athletes’ engagement and burnout. It can be seen that as motivation increases, there is also a steady rise in engagement. The same is true of burnout, the sole difference being that in the former case the relationships are positive, except in the case of the relationship between amotivation and engagement, whilst in the latter case the relationships are negative, with the sole exception of the relationship between amotivation and burnout.

### 3.4. Engagement’s Role as a Mediator between Motivation and Burnout

**Figure 1:**
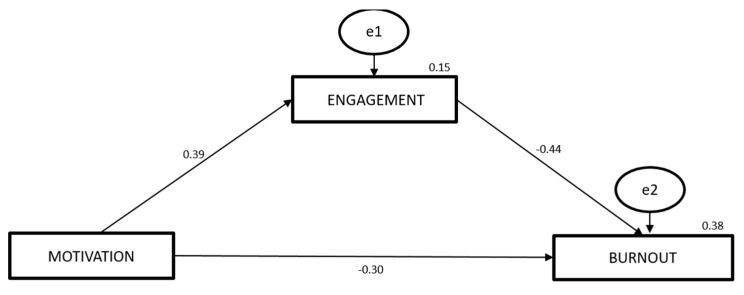
Model for the path analysis between motivation, engagement, and burnout.

Figure 1 shows the path analysis model specifying the mediating role of engagement in the relationship between motivation and the symptoms of burnout experienced by athletes. The numbers above the arrows indicate the standardized estimates on a 0−1 scale for the direct effects of motivation on burnout (−0.30, *p* < 0.001), motivation on engagement (0.39, *p* < 0.001), and engagement on burnout (−0.44, *p* < 0.001). All the direct effects occurred in the expected direction, and all registered statistical significance (*p* < 0.001).

Multiplying the direct effect of motivation on engagement (0.39) by the direct effect of engagement on burnout (−0.44) produces the indirect effect of motivation on burnout: −0.1716 (*p* < 0.001). In other words, motivation has two effects on burnout: one direct, −0.30, and a second indirect effect through engagement, −0.1716, bringing the total effect to −0.4716 (*p* < 0.001). Figure 1 also provides the proportion of variance to engagement attributable to motivation (R^2^ = 0.15) and the proportion of total variance to burnout attributable to motivation and engagement (R^2^ = 0.38).

### 3.5. Effect Comparison by Gender and Sport Type

During a second phase, comparisons were made between the direct effects of male (n_1_ = 370) and female athletes (n_2_ = 130) and between athletes participating in collective (n_1_ = 328) and individual (n_2_ = 172) sports. Table 3 gives the results of the initial comparison, which show that none of the differences analyzed reached statistical significance (*p* > 0.05).

However, in the case of athletes participating in collective and individual sports (Table 4), statistical significance was observed in the difference between the coefficient that reflects the effect of motivation on engagement in team athletes (0.32) and the same coefficient for individual athletes (0.54), with a value of Z = −3.35 (*p* < 0.001).

## 4. Discussion

The main objective of this study was to analyze the relationship between motivation and burnout, as well as the mediating role of engagement between these two constructs in a sample of Spanish athletes. In order to do this, several hypotheses were tested: those posed in the SDT [1,5] and in the work of Vallerand [25,37], proposing positive relationships between motivation and engagement and negative relationships between motivation and burnout and between engagement and burnout. In line with the model proposed in this study, the greater the self-determination of athletes’ motivation, the greater the probability of observing engagement and the lower the likelihood of the presence of sport burnout symptoms.

### 4.1. The Relationship between Motivation, Engagement, and Burnout

The results of our study are in line with all the hypotheses tested. As the level of specification in order to obtain in-depth insight into the relationship between each level of motivation with the other two variables was high, in the case of the path analysis, the objective was to come up with a simple model that would shed light on the fundamental role that motivation and engagement play on burnout within the context of sport.

As in other recent research projects [38,39], a significant positive relationship was found between motivation and engagement, although the three studies used different instruments to measure the motivation variable. Specifically, the model shows that 15% of the variance of engagement can be explained through motivation, compared with 15% and 21% in the aforementioned studies. It is reasonable to assume that this result is attributable to the fact that athletes’ motivation levels, which range from self-determined motivation to amotivation, influence their degree of confidence, the amount of time and effort they dedicate to sport, their physical and mental vigor, and their enthusiasm and enjoyment of sport.

As for the negative relationship between motivation and burnout, this was also shown to be significant, in line with the hypothesis proposed. Furthermore, in the case of our model, motivation accounted for 23% of the variance of burnout (see Figure 1). The results of previous research support these findings, where the same relationship was found and where motivation could explain significant proportions of the burnout variable [39,40]. Indeed, a review of this question led to the conclusion that burnout and its three dimensions are negatively influenced by extrinsic and intrinsic motivation and positively in the case of amotivation [41]. This may be explained by athletes’ perception that their motivation to practice is attributable to a series of stimuli, either internal (such as the desire to perfect their ability, learn and explore, etc.) or external (awards, recognition from others in their circle, avoiding punishment, remuneration, etc.). 

As was to be expected, the relationship between engagement and burnout, which have traditionally been considered opposing concepts, was found to be both significant and negative. In this sense, previous studies have considered engagement to be a suitable method in the prevention of burnout [30,42], and our results are in line with these findings [32,43]; in the context of the work, these same concepts have also been proven empirically, indicating a strong, inverse relationship [14].

In the case of this study, all the levels of motivation included in the SMS showed a significant relationship with engagement and burnout, with the exception of external regulation and burnout, which failed to reach the required level of significance. Our contribution is innovative given the degree of detail included in the comparisons between motivation and engagement and between motivation and burnout, which were conducted for all seven levels of motivation described in the SMS.

In a recent study [44] the relationships between these three variables and the sense of community has been analyzed but without deepening the mediating role of one of them. The proposed model also revealed the direct effects of motivation on burnout and the mediating role of engagement between these two factors, allowing for an explanation of more than a third of the variance of burnout through the twofold influence of motivation.

### 4.2. Gender and Sport Type

Although one of the limitations of the study is that women comprise a mere 26% of the sample, comparisons of the effects between male and female athletes were drawn in order to observe potential differences between motivation on burnout, motivation on engagement, and engagement on burnout. No significant differences were noted, indicating a measurement invariance of engagement in line with an earlier study that compared the participants’ competition levels [32]. Other research projects have yielded varying results in terms of motivation and gender [45]. The findings showed that women tend to display a more intrinsic motivation than men [46,47,48]. This contribution is innovative, as although the findings indicate differences in the degree of motivation between sexes, they do not affect the proposed model. The world of sport would benefit from further research that takes into consideration the differences and similarities between men and women in order to obtain in-depth insight into the gender-inherent characteristics that influence the degree of self-determination in motivation, in addition to other aspects such as conciliation, post-maternity professional practice, etc., situations that contribute to a greater understanding of athletes’ circumstances.

A final point for consideration is the difference observed between individual and collective sports. As observed in previous research, the outcomes indicate that the effect of motivation on engagement is considerably greater in the case of individual athletes (0.54) than those practicing collective sports (0.32), meaning that modifications in athletes’ motivation will lead to greater effects on engagement in the context of individual sports [49]. This difference may be due to the fact that a high degree of motivation—in other words, intrinsic motivation—is attributable to the athletes’ internal, more personal reasons. In the case of collective sports, this motivation may be the result of other factors, such as the sense of belonging to a group, which does not generate confidence, dedication, vigor, and enthusiasm in such an intimate and personalized manner as in the case of individual sports. Other possible explanations may be related to factors such as more accessible decision making for individual athletes or personalized feedback.

### 4.3. Limitations and Potential of the Study

Among the limitations of the study, it is necessary to comment on the use of a non-probabilistic sampling procedure, the age range of the athletes, between 12 and 29 years old, and the greater number of male than female athletes present in the sample. Moreover, it should be taken into account that over time there may be changes in the relationships studied in athletes. It would be advisable to assess the design of a longitudinal study that could consolidate the results by offering data on the stability of the variances throughout the professional development of athletes.

Finally, the present research tries to take a novel perspective by proposing motivation as a mediator variable between engagement and burnout, while the relationship of each of the seven types of motivation and their effects on burnout and engagement is analyzed. In addition, the actual investigation aims to be a new test for the invariance of the measures analyzed through the gender of the athletes.

## 5. Conclusions

This study confirms the hypotheses formulated by the SDT and extends its validity within the field of sport psychology. The practical implications of the results shed further light on the relationship between some of the psychological processes involved in professional athletes’ performance: a high level of self-determined motivation is associated with athletes whose enjoyment stems from the mere fact of participating in their activity, which in itself contributes to the prevention of burnout. However, in accordance with the model presented here, promoting engagement in order to reduce burnout has emerged as a key factor for consideration. In light of this inverse relationship—namely that the greater the degree of confidence, vigor, dedication, and enthusiasm, the less likely burnout is to occur, and the level of satisfaction and permanence in the sport will rise—future studies should extend this initial work where engagement is presented as a mediator between motivation and burnout.

## Figures and Tables

**Table 1:** Descriptive statistics.

	Mean	Standard Deviation	Skewness	Kurtosis
IM to experience stimulation	5.38	1.20	−0.74	0.13
IM to accomplish things	5.10	1.31	−0.61	−0.10
IM to know	5.05	1.35	−0.61	−0.12
Identified regulation	4.59	1.39	−0.29	−0.48
Introjected regulation	4.55	1.34	−0.28	−0.34
External regulation	3.78	1.53	0.04	−0.78
Amotivation	2.81	1.19	0.79	0.56
Motivation (total)	5.17	3.85	−0.27	0.00
Confidence	3.75	0.73	−0.32	−0.13
Vigour	4.12	0.67	−0.65	0.25
Dedication	4.16	0.77	−1.03	0.81
Enthusiasm	4.60	0.57	−1.93	4.68
Engagement (total)	4.16	0.55	−0.86	0.92
PEE	2.54	1.49	1.28	1.42
SD	1.71	0.82	1.32	1.15
RSA	2.21	0.69	0.38	−0.02
Burnout(total)	2.15	0.73	1.02	1.78

IM: intrinsic motivation; PEE: physical and emotional exhaustion; SD: sport devaluation; RSA: reduced sense of accomplishment.

**Table 2:** Relationship between motivation levels and engagement and burnout.

	Engagement	Burnout
IM to experience stimulation	0.61 ***	−0.41 ***
IM to accomplish things	0.54 ***	−0.34 ***
IM to know	0.58 ***	−0.36 ***
Identified regulation	0.26 ***	−0.17 ***
Introjected regulation	0.30 ***	−0.11 ***
External regulation	0.20 ***	−0.04
Amotivation	−0.19 ***	0.36 ***

*** *p* < 0.001.

**Table 3:** Comparison of effects between male and female athletes.

Effect	Male Athletes	Female Athletes	Z	*p*
Motivation on burnout	−0.29	−0.33	−0.52	>0.05
Motivation on engagement	0.35	0.53	−1.70	>0.05
Engagement on burnout	−0.44	−0.41	0.02	>0.05

**Table 4:** Comparison of effects between athletes participating in team and individual sports.

Effect	Team Athletes	Individual Athletes	Z	*p*
Motivation on burnout	−0.31	−0.30	0.28	>0.05
Motivation on engagement	0.32	0.54	−3.35	<0.001
Engagement on burnout	−0.44	−0.41	0.58	>0.05
